# Metabolic Activity of Visceral Adipose Tissue Is Associated with Metastatic Status of Lymph Nodes in Endometrial Cancer: A ^18^F-FDG PET/CT Study

**DOI:** 10.3390/ijerph19010092

**Published:** 2021-12-22

**Authors:** Kisoo Pahk, Ki-Jin Ryu, Chanmin Joung, Hyun Woo Kwon, Sanghoon Lee, Hyuntae Park, Tak Kim, Jae Yun Song, Sungeun Kim

**Affiliations:** 1 Department of Nuclear Medicine, Korea University Anam Hospital, Seoul 02841, Korea; kisu99@korea.ac.kr (K.P.); hnwoo@korea.ac.kr (H.W.K.); 2 Department of Obstetrics and Gynecology, Korea University Anam Hospital, Seoul 02841, Korea; ryukj@korea.ac.kr (K.-J.R.); mdleesh@korea.ac.kr (S.L.); cyberpelvi@korea.ac.kr (H.P.); tkim@kumc.or.kr (T.K.); 3 Institute of Inflammation Control, Korea University, Seoul 02841, Korea; joungchanmin@korea.ac.kr

**Keywords:** obesity, visceral fat, endometrial cancer, metastasis, inflammation, positron-emission tomography

## Abstract

Obesity contributes to increased cancer incidence and aggressiveness in patients with endometrial cancer. Inflamed metabolic activity of visceral adipose tissue (VAT) is regarded as a key underlying mechanism of adverse consequences of obesity. The aim of this study was to investigate the association between inflammatory metabolic activity of VAT evaluated by ^18^F-fluorodeoxyglucose positron emission tomography/computed tomography (^18^F-FDG PET/CT) and metastatic status of lymph nodes (LN) in patients with endometrial cancer. In total, 161 women with newly diagnosed endometrial cancer, who received preoperative 18F-FDG PET/CT, were enrolled. VAT inflammatory metabolic activity was defined as V/S ratio and measured from the maximum standardized uptake value (SUVmax) of VAT normalized to the SUVmax of subcutaneous adipose tissue (SAT). The positive LN metastasis group exhibited a significantly higher V/S ratio than the negative LN metastasis group. Systemic inflammatory surrogate markers including high sensitivity C-reactive protein, spleen SUVmax, and bone marrow SUVmax were also higher in the LN metastasis group than in the negative LN metastasis group, showing significant correlations with V/S ratio. In multivariate logistic regression analysis, V/S ratio was independently associated with LN metastasis. V/S ratio is independently associated with the LN metastasis status in patients with endometrial cancer. This finding could be useful as a potential surrogate marker of obesity-induced VAT inflammation associated with tumor aggressiveness.

## 1. Introduction

Obesity is an important public health issue today. It affects more than 600 million people globally. Its prevalence is higher than 36% in the United States [1,2]. Furthermore, its prevalence is continuously increasing in both developing and developed countries [3].

Endometrial cancer is the most common gynecologic cancer in developed countries. Its prevalence is also continuously increasing worldwide [4]. Accumulating evidences have suggested that obesity elevates cancer risk and tumor aggressiveness, which escalate both morbidity and mortality of endometrial cancer patients [5,6,7]. Considering the causal relationship between obesity and endometrial cancer, one crucial pathophysiological mechanism is related to obesity-induced inflammatory metabolic activity of visceral adipose tissue (VAT) that supports tumorigenesis and tumor progression [8,9]. Inflamed VAT can secrete an array of pro-inflammatory cytokines, such as interleukin-6 (IL-6) and tumor necrosis factor-alpha (TNF-α), thereby stimulating tumor-associated macrophage infiltration and upregulating cancer stem cells, which collectively contribute to the development of a pro-tumorigenic environment [8,9].

Lymph node (LN) metastasis is a well-known key factor for determining the prognostic outcome and planning optimized treatment strategies in patients with endometrial cancer [10,11]. Furthermore, Ye et al. [12] have reported that visceral obesity is related to positive LN metastasis in patients with endometrial cancer. However, detailed underlying mechanisms that translate metabolic activity of inflamed VAT into LN metastasis in endometrial cancer remains to be fully explored.

^18^F-fluorodeoxyglucose positron emission tomography/computed tomography (^18^F-FDG PET/CT) is a well-known imaging modality to measure VAT metabolic activity, non-invasively [13,14,15,16]. ^18^F-FDG PET/CT can reflect the inflammatory activity of classically activated (M1) macrophage, which is increased in obesity-driven inflamed VAT [17,18]. Furthermore, numerous recent studies have found that upregulated VAT metabolic activity evaluated by ^18^F-FDG PET/CT is associated with tumor aggressiveness in breast, colorectal, and thyroid cancer, for which obesity is a major risk factor [14,15,16]. Based on these findings, the present study hypothesized that increased VAT metabolic activity could also be associated with LN metastasis in endometrial cancer. Thus, the aim of this study was to explore whether VAT metabolic activity evaluated by ^18^F-FDG PET/CT might be related to LN metastasis and could be used as a potential surrogate marker of obesity-induced VAT inflammation in endometrial cancer.

## 2. Materials and Methods

### 2.1. Study Patients

**Figure 1:**
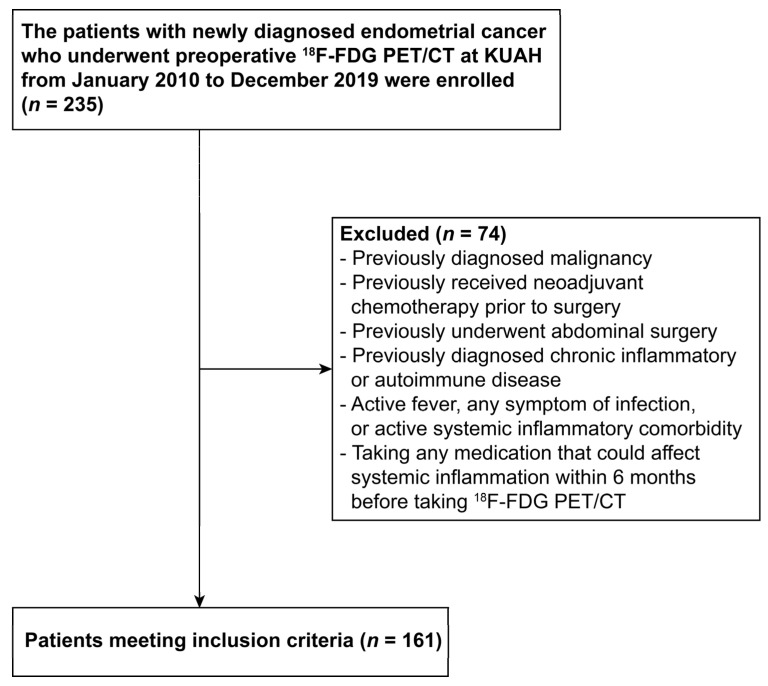
Flow chart showing the study design. ^18^F-FDG PET/CT: ^18^F-fluorodeoxyglucose positron emission tomography/computed tomography; KUAH: Korea University Anam Hospital.

A total of 235 patients with newly diagnosed endometrial cancer, who received preoperative ^18^F-FDG PET/CT and surgery from January 2010 to December 2019 were enrolled in this study, retrospectively. Exclusion criteria are presented in the flow chart (Figure 1). Finally, a total of 161 patients were enrolled in this study. This study was approved by the Institutional Review Board of Korea University Anam Hospital (Approval No. 2020AN0564). The requirement of informed consent was waived by the Institutional Review Board due to the retrospective nature of this study.

### 2.2. Anthropometric and Laboratory Measurements

Body mass index (BMI) was calculated as weight/height squared (kg/m^2^). All blood samples were acquired within one week before surgery. The levels of high-sensitivity C-reactive protein (hsCRP) were analyzed by using a chemiluminescence immunoassay (Beckman Coulter, Brea, CA, USA). Serologic tumor markers including carcinoembryonic antigen (CEA), carbohydrate antigen 19-9 (CA 19-9), and carbohydrate antigen 125 (CA 125) were determined by radioimmunoassay (Gamma Pro, Kaien, Seoul, Korea). 

### 2.3. ^18^F-FDG PET/CT Protocol

All patients fasted overnight prior to taking ^18^F-FDG PET/CT. Image acquisition range was covered from the vertex of the skull to the proximal thigh at one hour after intravenous administration of 5.29 MBq/kg ^18^F-FDG using a dedicated PET/CT scanner (Gemini TF, Philips Medical Systems, Cleveland, OH, USA). For attenuation correction, a non-contrast-enhanced CT scan (120 kVp, 50 mA, 4 mm slice thickness) was taken first and a subsequent PET scan was performed. PET scans were taken for 9 bed positions with a scan duration time of 1 min per bed position. Image reconstruction was performed iteratively using a three-dimensional row-action maximum likelihood algorithm.

### 2.4. Image Analysis 

Images were analyzed by two experienced nuclear medicine radiologists (KP and HWK) using a commercially available workstation (Extended Brilliance Workspace version 3.5, Philips Healthcare, Eindhoven, Netherlands). They were blinded to patients’ clinical information.

For image evaluation, based on predefined Hounsfield units (range −70 to −110) on CT images, both VAT and subcutaneous adipose tissue (SAT) were recognized, as previously mentioned [13,14,15,16]. Next, a region of interest (ROI) was placed, and standardized uptake value (SUV) was calculated as follows:

SUV = Tracer activity (ROI) (MBq/mL)/Injected dose (MBq)/Total body weight (g)

For the evaluation of VAT metabolic activity, ROIs were placed along the boundaries of intra-abdominal fat on three consecutive axial slices (between the level of L4 and L5 vertebrae) and adjusted to exclude overspill ^18^F-FDG activity in the vessel, muscle, and/or intestine, as previously described [14,15,16]. Maximum SUVs from three consecutive ROIs were acquired and an averaged maximum SUV from those ROIs was defined as VAT SUVmax. For the evaluation of SAT metabolic activity, ROIs were also placed on the buttock or anterior abdominal wall area on three consecutive axial slices, as previously described [14,15,16]. These maximum SUVs from three consecutive ROIs were acquired and the averaged maximum SUV from those ROIs was defined as SAT SUVmax. VAT metabolic activity was defined as VAT/SAT (V/S) ratio, which was calculated as follows:

V/S ratio = VAT SUVmax/SAT SUVmax

Increased metabolic activity of both spleen and bone marrow (BM) evaluated by ^18^F-FDG PET/CT is known to reflect elevated myeloid activity, which is associated with increased systemic inflammation, and can thereby be used as surrogate markers of systemic inflammation [19,20]. For the evaluation of metabolic activity of both spleen and BM, ROIs were placed on the spleen and BM of L3 to L5 vertebrae, as previously described [20]. Averaged SUVmax from those ROIs from all axial slices were designated as spleen, and BM SUVmax, respectively [20]. In the present study, both intra- and interobserver correlation analyses on measured SUVs exhibited excellent reliability (correlation coefficient > 0.9).

### 2.5. Surgery and Pathology

All patients received a total abdominal hysterectomy, bilateral salpingo-oophorectomy, and pelvic-, and para-aortic lymphadenectomy according to the routine staging surgery protocol in our institution. Final pathologic diagnosis was confirmed by examining surgical specimens. Patients were staged according to the International Federation of Gynecology and Obstetrics (FIGO) 2014 staging for endometrial cancer [21]. Histologic grade was performed using a three-tier grading system based on the degree of glandular formation (grade 1: well-differentiated; grade 2: moderately differentiated; grade 3: poorly differentiated) [22]. Non-endometrioid subtypes such as serous, clear cell, and carcinosarcoma were considered as grade 3. 

### 2.6. Statistical Analysis

All data are exhibited as mean ± standard deviation. For the analysis of categorical variables, a Chi-squared (χ2) test or Fisher’s exact test was employed. Shapiro-Wilk was used to determine the normalcy of continuous variables and Student’s t-test, or Mann-Whitney U test was performed. Spearman’s correlation coefficient, receiver-operating characteristic (ROC) curve analysis, and logistic regression analysis were also employed as statistical methods. All data were analyzed using MedCalc software version 18.1 (MedCalc, Mariakerke, Belgium) and SPSS software version 17.0 (SPSS Inc., Chicago, IL, USA). A *p*-value less than 0.05 was considered statistically significant.

## 3. Results

Of a total of 161 endometrial cancer patients, 24 were confirmed to have LN metastasis, while 137 had no LN metastasis. Patients with positive LN metastasis showed significantly increased systemic inflammation and more aggressive tumor characteristics than those without LN metastasis. Clinical characteristics of all patients are presented in Table 1. 

### 3.1. VAT Metabolic Activity Is Increased in Endometrial Cancer Patients with LN Metastasis 

**Figure 2:**
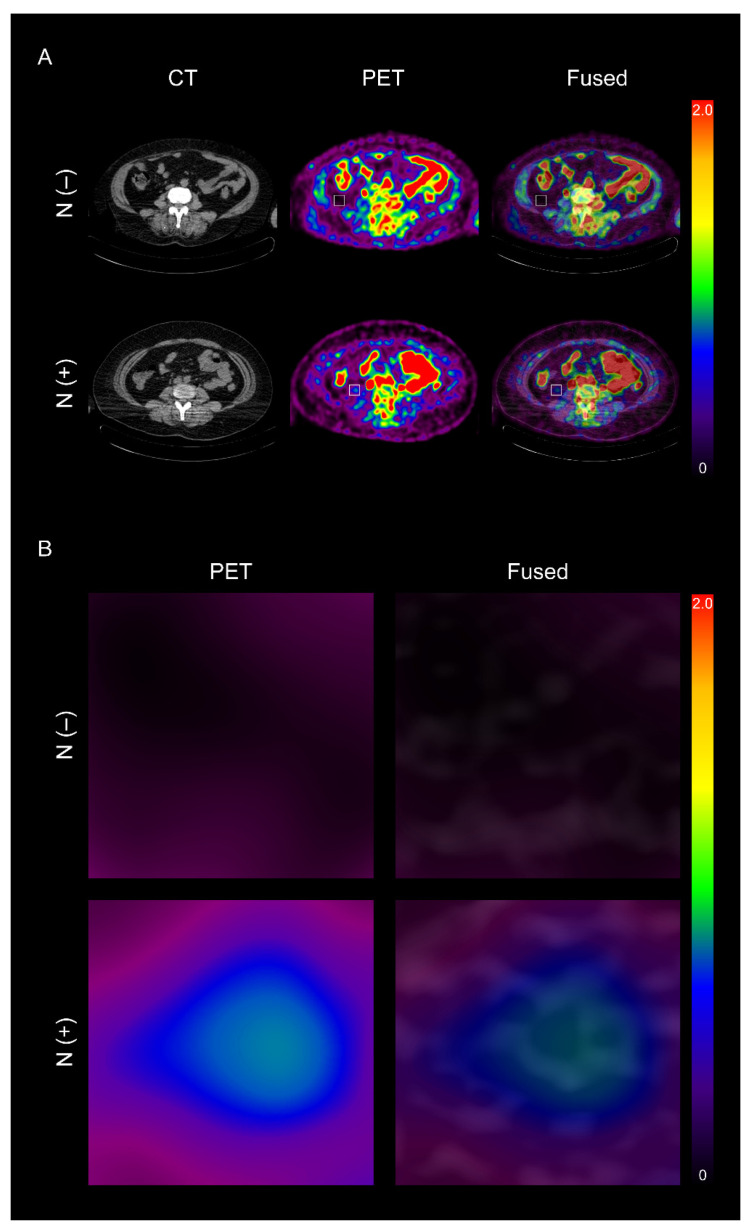
Representative ^18^F-FDG PET/CT images of visceral adipose tissue (VAT) metabolic activity according to lymph node (LN) metastasis status (**A**) and its corresponding magnified images of VAT (**B**). N (−): negative LN metastasis, N (+): positive LN metastasis, ^18^F-FDG: ^18^F-fluorodeoxyglucose, PET: positron emission tomography, CT: computed tomography.

**Figure 3:**
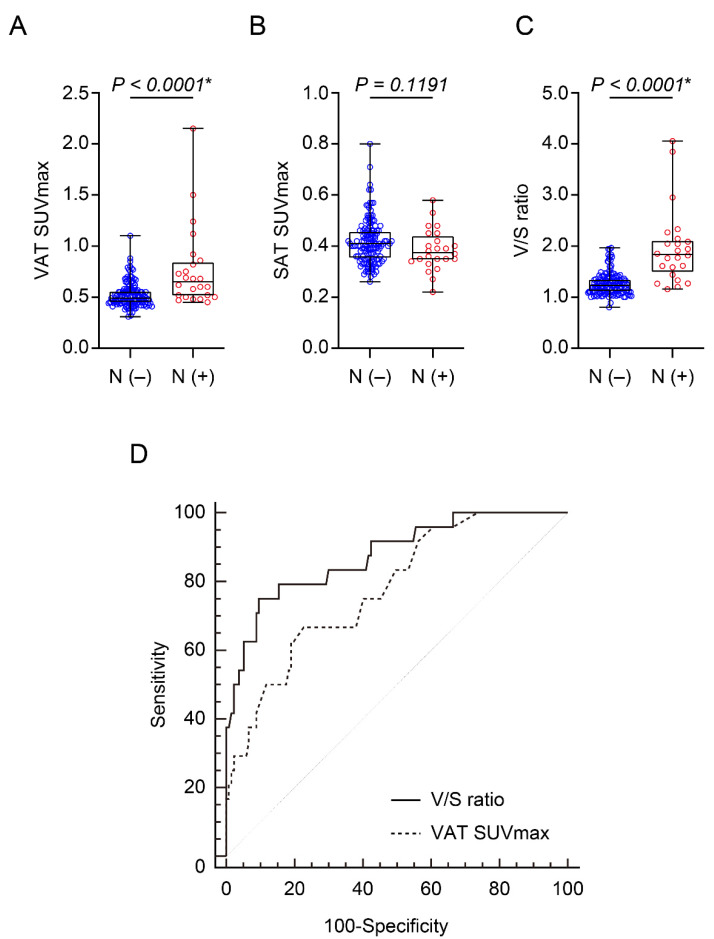
Comparison of: (**A**) VAT SUVmax, (**B**) SAT SUVmax, and (**C**) V/S ratio according to LN metastasis status in patients with endometrial cancer. (**D**) Receiver operating characteristic (ROC) curve analysis for the prediction of LN metastasis. N (−), *n* = 137; N (+), *n* = 24. SUVmax: standardized uptake value, SAT: subcutaneous adipose tissue, V/S ratio: VAT SUVmax/SAT SUVmax, * statistically significant difference.

Whether VAT metabolic activity was upregulated in patients with LN metastasis was first investigated. As shown in Figure 2 and Figure 3, the positive LN metastasis group presented significantly higher VAT SUVmax (0.77 ± 0.39 vs. 0.52 ± 0.12, *p* < 0.0001, Figure 3A) and V/S ratio (1.97 ± 0.74 vs. 1.27 ± 0.22, *p* < 0.0001, Figure 3C) than those without LN metastasis. In contrast, SAT SUVmax presented no significant difference between the two groups (Figure 3B).

### 3.2. Relationship between VAT Metabolic Activity and Systemic Inflammation

Established systemic inflammation surrogate markers such as spleen, BM SUVmax, and hsCRP [19,20], were significantly higher in endometrial cancer patients with LN metastasis than in those without LN metastasis (Table 1). Thus, we next examined whether VAT SUVmax showed a significant correlation with systemic inflammation in patients with endometrial cancer. As shown in Table 2, both VAT SUVmax and V/S ratio were significantly correlated with surrogate markers for systemic inflammation, consistent with previous studies [23,24]. In contrast, SAT SUVmax was not significantly correlated with systemic inflammation. 

### 3.3. VAT Metabolic Activity Is Independently Associated with LN Metastasis in Patients with Endometrial Cancer

According to ROC curve analysis, as shown in Figure 3D, the optimal cut-off values of V/S ratio and VAT SUVmax to identify LN metastasis were 1.5667 and 0.57, respectively. Area under the curve (AUC) for the optimal cut-off value of V/S ratio was 0.877 (95% confidence interval 0.816–0.923; standard error 0.0415; *p* < 0.0001). The sensitivity and specificity were 75% and 90.5%, respectively. AUC was 0.777 (95% confidence interval 0.705–0.839; standard error 0.0502; *p* < 0.0001) for VAT SUVmax. The sensitivity and specificity were 66.7% and 77.4%, respectively. Furthermore, statistically significant higher AUC was observed for V/S ratio compared to VAT SUVmax (*p* = 0.0212). Thus, these findings suggest that V/S ratio could be more suitable for evaluation of LN metastasis in endometrial cancer patients than VAT SUVmax.

Next, using the optimal cut-off value of V/S ratio, we further performed univariate-and multivariate logistic regression analyses to evaluate the association between V/S ratio and LN metastasis. Univariate analysis showed that non-endometrioid histology, high histologic grade, large tumor size, positive lymphovascular invasion, positive ovary and/or salphinx involvement, and high V/S ratio were significantly associated with positive LN metastasis (Table 3). In multivariate regression analysis, V/S ratio was independently associated with positive LN metastasis with the highest odds ratio (Table 3).

## 4. Discussion

To our knowledge, this is the first human study to explore the association between the metabolic activity of VAT and metastatic status of the LN in patients with endometrial cancer using preoperative ^18^F-FDG PET/CT. In this study, we found that VAT metabolic activity, defined as V/S ratio, was elevated in patients with LN metastasis and showed a positive correlation with systemic inflammation surrogate markers. Furthermore, it was independently associated with positive LN metastasis even after adjusting other known risk factors.

Inflamed VAT is a major contributor to the progression of systemic inflammation by secreting numerous pro-inflammatory cytokines including IL-1β, IL-6, and TNF-α into systemic circulation, which could have a remote effect leading to an increase in tumor aggressiveness [8,9]. In this study, we found that both VAT metabolic activity defined as V/S ratio and systemic inflammation were increased in patients with LN metastasis and that V/S ratio showed significant correlation with systemic inflammation. Collectively, these findings provide evidence that V/S ratio can reflect the inflammatory burden from VAT, which affects systemic inflammation, thereby leading to enhanced tumor aggressiveness. 

BMI, a classical anthropometric measurement, is easily obtainable. It has been widely used to evaluate the relationship between obesity and endometrial cancer in numerous previous studies [5,6,7]. However, the Women’s Health Initiative study has reported that BMI is not fully sufficient as an obesity surrogate marker in assessing the relationship between obesity and stage or grade of endometrial cancer [25]. Consistent with previous findings, we also observed that there was no significant difference in BMI between patients with LN metastasis and those without LN metastasis in the present study (Table 1). Furthermore, BMI was not significantly associated with LN metastasis in patients with endometrial cancer (Table 3). BMI cannot fully capture the inflamed VAT activity, a key underlying pathological mechanism that drives clinical and metabolic consequences of obesity [26,27]. Therefore, based on our study, we believe that metabolic activity of VAT, which was measured as V/S ratio, might serve as a surrogate marker of tumor aggressiveness, which is related to obesity-driven inflamed VAT activity in endometrial cancer patients in addition to BMI. 

In the clinical decision process for endometrial cancer patients, lymphadenectomy has been widely used for surgical staging and it can often be associated with major comorbidities such as genitofemoral nerve injury, lymphoedema, and lymphocyst formation [28,29,30]. To minimize the adverse effect of lymphadenectomy, some surgeons have suggested that a comprehensive lymphadenectomy should be performed in patients with high-risk characteristics for LN metastasis such as high grade and large primary tumors [31], which were consistent with our results. Notably, we found that the V/S ratio was associated with LN metastasis with the highest odds ratio among known high-risk features for LN metastasis. Thus, V/S ratio can be incorporated into algorithms to select patients for lymphadenectomy, thereby avoiding unnecessary severe comorbidities. However, a further large prospective study is needed. 

Accumulating large prospective and epidemiologic studies have suggested that high-levels of physical activity against obesity can reduce the risk of endometrial cancer, whereas sedentary behavior can increase such risk [32,33,34]. Furthermore, recently, a prospective study by Friedenreich et al. [35] has observed that increased physical activity after a diagnosis of endometrial cancer is associated with better survival and suggested that health care professionals should recommend physical activity as a therapeutic intervention to patients with newly diagnosed endometrial cancer and endometrial cancer survivors to improve their survival outcomes. Given that inflamed VAT can be regarded as a causal mechanism of obesity-induced endometrial cancer, it is conceivable that V/S ratio evaluated by ^18^F-FDG PET/CT could be useful for assessing responses to therapeutic interventions for obesity such as exercise or lifestyle modification in patients with endometrial cancer. 

First, this study was conducted with a small sample size in a single center, which could induce a selection bias. Second, although extensive studies have shown that ^18^F-FDG PET/CT is suitable for assessment of VAT metabolic activity [13,14,15,16], we could not perform histological analysis on VAT tissue, which could further support our results. Lastly, we were unable to manage possible factors that might affect the distribution of ^18^F-FDG such as acquisition image time after tracer administration, plasma glucose levels, nor the insulin levels of patients. Nevertheless, findings of the present study counterbalanced these limitations by using a unique and functional imaging modality for the evaluation of VAT metabolic activity, to investigate the association between obesity-induced inflamed VAT metabolic activity and LN metastasis in endometrial cancer patients. 

## 5. Conclusions

Taken together, our results highlight that VAT metabolic activity, defined as V/S ratio, was independently associated with LN metastasis in patients with endometrial cancer and that it was synchronized with systemic inflammation. Thus, it can be used as a potential surrogate marker of obesity-induced VAT inflammation associated with tumor aggressiveness in endometrial cancer.

## Figures and Tables

**Table 1:** Patient characteristics.

	N (−)	N (+)	*p*
No. of patients	137	24	
Age (years)	55.4 ± 11.5	60.3 ± 7.5	0.047 *
BMI (kg/m^2^)	26.5 ± 4.8	25.1 ± 3.3	0.316
Menopause, n (%)			0.161
None	50 (36.5)	6 (25)	
Yes	87 (63.5)	18 (75)	
HTN, n (%)			0.981
None	91 (66.4)	16 (66.7)	
Yes	46 (33.6)	8 (33.3)	
DM, n (%)			1
None	116 (84.7)	21 (87.5)	
Yes	21 (15.3)	3 (12.5)	
Histology, n (%)			<0.001 *
Endometrioid-origin	116 (84.7)	12 (50)	
Other	21 (15.3)	12 (50)	
Histologic grade, n (%)			<0.001 *
1	75 (54.7)	4 (16.7)	
2	38 (27.7)	5 (20.8)	
3	24 (17.6)	15 (62.5)	
Tumor size (cm)	4.5 ± 2.9	7.1 ± 3.7	<0.001 *
FIGO stage, n (%)			<0.001 *
1	112 (81.8)	1 (4.2)	
2	15 (10.9)	3 (12.5)	
3	9 (6.6)	15 (62.5)	
4	1 (0.7)	5 (20.8)	
Ovary/salphinx involvement, n (%)			0.001 *
None	126 (92)	15 (62.5)	
Yes	11 (8)	9 (37.5)	
Lymphovascular invasion, n (%)			<0.001 *
None	115 (83.9)	10 (41.7)	
Yes	22 (16.1)	14 (58.3)	
hsCRP, mg/dL	5.1 ± 14.7	6.6 ± 9.5	0.0083 *
Spleen SUVmax	2.07 ± 0.34	2.26 ± 0.46	0.01 *
BM SUVmax	1.82 ± 0.38	2.27 ± 0.77	0.02 *
CEA, ng/mL	1.38 ± 1.46	3.14 ± 6.46	0.0178 *
CA 19-9, U/mL	86.37 ± 467	291.76 ± 543.16	0.0019 *
CA 125, U/mL	31.83 ± 116.18	164.57 ± 459.91	<0.001 *

N (−) negative lymph node metastasis, N (+) positive lymph node metastasis, BMI body mass index, HTN hypertension, DM diabetes mellitus, FIGO International Federation of Gynecology and Obstetrics, hsCRP high-sensitivity C-reactive protein, SUVmax maximum standardized uptake value, BM bone marrow, CEA carcinoembryonic antigen, CA 19-9 carbohydrate antigen 19-9, CA 125 carbohydrate antigen 125. * Statistically significant difference.

**Table 2:** Spearman correlation analysis.

	VAT SUVmax		SAT SUVmax		V/S Ratio	
	r	p	r	p	r	p
Spleen SUVmax	0.198	0.012 *	0.142	0.072	0.158	0.046 *
BM SUVmax	0.253	0.001 *	0.072	0.367	0.226	0.004 *
hsCRP	0.341	<0.001 *	0.148	0.079	0.211	0.012 *

VAT visceral adipose tissue, SAT subcutaneous adipose tissue, SUVmax maximum standardized uptake value, V/S ratio VAT SUVmax/SAT SUVmax, *BM* bone marrow, hsCRP high-sensitivity C-reactive protein. * Statistically significant difference.

**Table 3:** Uni- and multivariate analyses for prediction of lymph node metastasis in patients with endometrial cancer.

	Univariate		Multivariate	
Variable	OR (95% CI)	*p*	OR (95% CI)	*p*
Age (Continuous)	1.043 (1–1.088)	0.05		
BMI (Continuous)	0.927 (0.834–1.031)	0.162		
Menopause (None vs. Yes)	2.093 (0.732–5.983)	0.168		
HTN (None vs. Yes)	0.989 (0.394–2.481)	0.981		
DM (None vs. Yes)	0.789 (0.216–2.884)	0.72		
Histology (Non-endometrioid vs. Endometrioid)	0.181 (0.072–0.457)	<0.001 *	0.696 (0.138–3.502)	0.66
Histologic grade (1 and 2 vs. 3)	7.708 (3.021–19.67)	<0.001 *	2.714 (0.545–13.51)	0.223
Tumor size (Continuous)	1.236 (1.075–1.421)	0.003 *	1.11 (0.884–1.394)	0.367
Lymphovascular invasion (None vs. Yes)	7.127 (2.808–18.089)	<0.001 *	1.405 (0.289–6.831)	0.674
Ovary/salphinx involvement (None vs. Yes)	6.6 (2.353–18.514)	<0.001 *	3.263 (0.615–17.327)	0.165
V/S ratio (≤1.56 vs. >1.56)	28.615 (9.656–84.799)	<0.001 *	23.2 (5.318–101.211)	<0.001 *
CEA (Continuous)	1.179 (0.978–1.421)	0.085		
CA 19-9 (Continuous)	1 (1–1.001)	0.229		
CA 125 (Continuous)	1.002 (1–1.004)	0.061		

OR odds ratio, CI confidence interval, BMI body mass index, HTN hypertension, DM diabetes mellitus, V/S ratio VAT SUVmax/SAT SUVmax, CEA carcinoembryonic antigen, CA 19-9 carbohydrate antigen 19-9, CA 125 carbohydrate antigen 125. * Statistically significant difference.

## Data Availability

The data presented in this study are available on request from the corresponding author. The data are not publicly available due to privacy and ethical reasons.
